# Progressive Superior Sagittal Sinus Thrombosis Successfully Treated With Angioplasty: A Case Report

**DOI:** 10.7759/cureus.91517

**Published:** 2025-09-03

**Authors:** Chie Matsuura, Yuki Sakaeyama, Yutaka Fuchinoue, Sayaka Terazono, Nobuo Sugo

**Affiliations:** 1 Department of Neurosurgery, Faculty of Medicine, Toho University, Tokyo, JPN

**Keywords:** balloon angioplasty, endovascular therapy, sinus thrombosis, subarachnoid hemorrhage, superior sagittal sinus

## Abstract

Although anticoagulation is the standard treatment for cerebral venous sinus thrombosis (CVST), the efficacy of endovascular therapy remains unproven. Medical treatment requires time to show effectiveness, and some patients may have a poor prognosis. Here, we report a case of progressively worsening superior sagittal sinus thrombosis successfully treated with balloon angioplasty, resulting in a favorable outcome. A 68-year-old man presented with right upper limb weakness and aphasia. Head CT revealed a subarachnoid hemorrhage in the left frontal region. Cerebral angiography confirmed the diagnosis of superior sagittal sinus thrombosis. Despite heparin infusion therapy, the patient developed hemorrhagic infarction in the left frontal lobe on day 3 and in the right parietal lobe on day 7. As the patient’s level of consciousness deteriorated progressively, balloon angioplasty was performed on day 10 to address stenosis of the superior sagittal sinus. After treatment, the patient showed no further hemorrhage and experienced amelioration in consciousness. Thereafter, anticoagulant treatment was switched to oral warfarin, and the patient was transferred to a rehabilitation facility with residual right hemiparesis and a modified Rankin Scale (mRS) score of three. In conclusion, balloon angioplasty may be a viable therapeutic option for progressive CVST.

## Introduction

Acute cerebral venous sinus thrombosis (CVST) accounts for approximately 0.5-3% of all strokes [1]. It occurs more frequently in women aged <55 years, and headache is noted in approximately 90% of cases [2]. The mainstay of treatment for CVST is medical therapy [2], although European Stroke Organization guidelines do not currently recommend endovascular treatment for this condition [3]. Conversely, medical treatment may require time to demonstrate efficacy, and certain cases may have an unfavorable prognosis. Accordingly, American Heart Association guidelines endorse endovascular therapy as a rescue treatment in cases with worsening symptoms or when standard therapy cannot be administered [2]. Thus, the efficacy and safety of endovascular treatment for CVST remain subjects of ongoing debate.

Several case reports have described successful endovascular interventions, including mechanical thrombectomy and balloon angioplasty, in patients with severe or refractory CVST [4,5]. These reports suggest that endovascular management may be a viable option in select cases.

In this report, we present a case of superior sagittal sinus thrombosis with progressively worsening neurological symptoms and radiological findings, in which balloon angioplasty was performed, resulting in a favorable outcome.

This article was previously presented at the 50th Annual Meeting of the Japan Stroke Society on March 6, 2025.

## Case presentation

**Figure 1:**
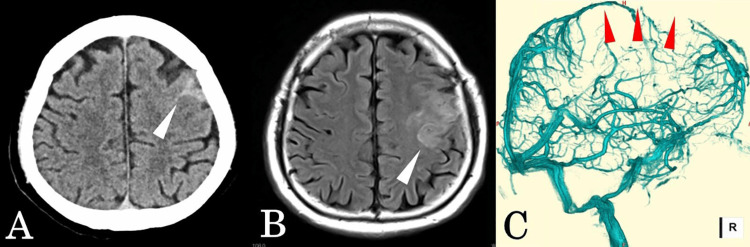
Imaging findings on admission (A) Head CT showing subarachnoid hemorrhage in the left frontal region (white arrowhead). (B) Head MRI showing high signal intensity in the left frontal lobe on FLAIR imaging (white arrowhead). (C) Cerebral angiography in the venous phase showing non-visualization of part of the superior sagittal sinus (red arrowhead) with delayed cortical venous drainage. FLAIR, fluid attenuated inversion recovery

A 68-year-old man presented with a medical history of hepatitis B carrier, no history of COVID-19 infection, and no recent vaccination. He was a non-smoker and an occasional alcohol consumer. He noticed right upper limb weakness upon waking and presented to our hospital on foot. His blood pressure was 152/86 mmHg, and his heart rate was 68 beats per minute. His level of consciousness was E4V1M6 on the Glasgow Coma Scale. Motor aphasia was noted along with right upper limb weakness graded as 4/5 on manual muscle testing. General laboratory findings are shown in Table 1. In addition, protein C activity, protein S activity, anti-platelet factor 4 antibody, anti-protease 3 antibody, anti-neutrophil cytoplasmic antibody, myeloperoxidase, and anticardiolipin IgG were all within normal limits. Head CT revealed subarachnoid hemorrhage in the left frontal region (Figure 1A). Head MRI revealed high signal intensity in the left frontal lobe on fluid attenuated inversion recovery (FLAIR) imaging (Figure 1B). Nonetheless, at admission, cerebral angiography showed normal findings in the arterial phase, although part of the superior sagittal sinus was not visualized, and delayed cortical venous drainage was observed in the venous phase (Figure 1C).

**Table 1:** Patient’s laboratory findings on admission AST: aspartate aminotransferase, ALT: alanine aminotransferase, HbA1c: hemoglobin A1c, PT-INR: prothrombin time-international normalized ratio, APTT: activated partial thromboplastin time

Laboratory findings	
C-reactive protein (mg/dL)	0.9
Sodium (mmol/L)	145
Potassium (mmol/L)	4.7
Total protein (g/dL)	7.5
Albumin (g/dL)	4.3
Urea nitrogen (mg/dL)	15
Creatinine (mg/dL)	1.06
AST (U/L)	23
ALT (U/L)	14
Glucose (mg/dL)	99
HbA1c (%)	5.4
White blood cell (10^3^/µL)	6.0
Red blood cell (10^3^/µL)	508
Hemoglobin (g/dL)	15.6
Platelet (10^4^/µL)	16.3
PT-INR	0.8
APTT (sec)	29.3
D-dimer (µg/mL)	2.1
Urine glucose	(-)
Urine protein	(-)
Urine occult blood	(-)
Urine-specific gravity	>1.035

**Figure 2:**
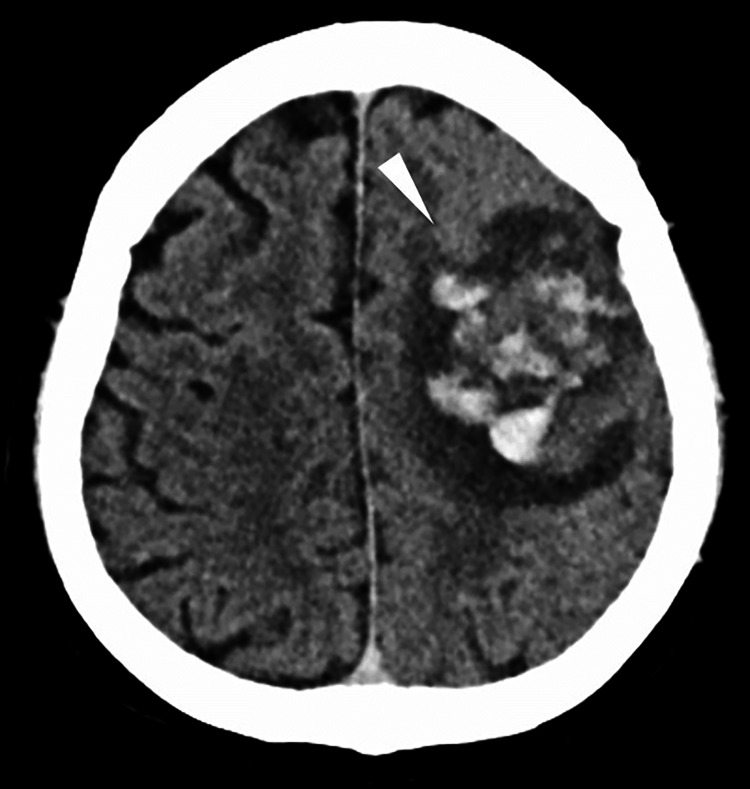
CT three days after admission Image showing intracerebral hemorrhage in the left frontal lobe (white arrowhead).

**Figure 3:**
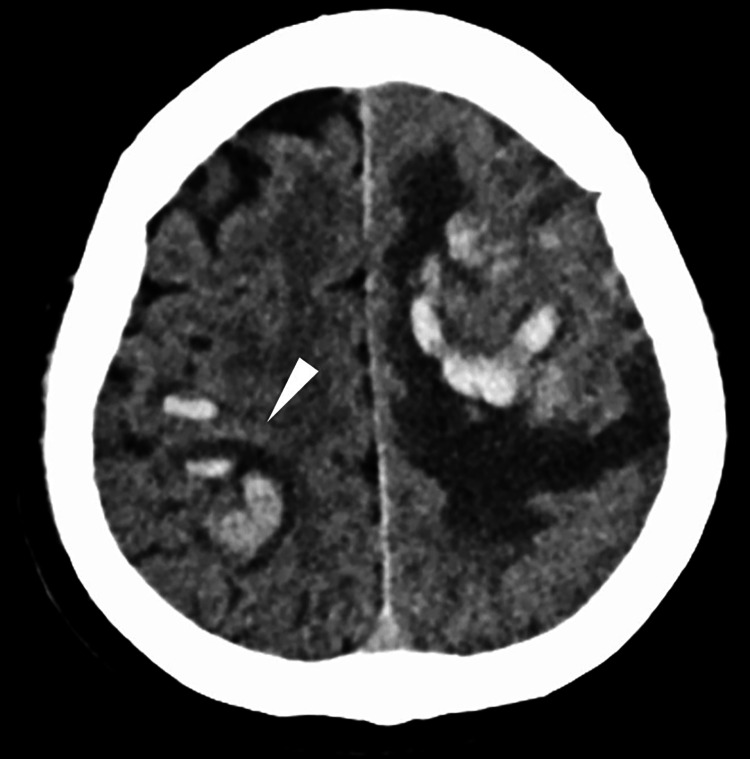
CT seven days after admission Image showing a new intracerebral hemorrhage in the right parietal lobe (white arrowhead).

**Figure 4:**
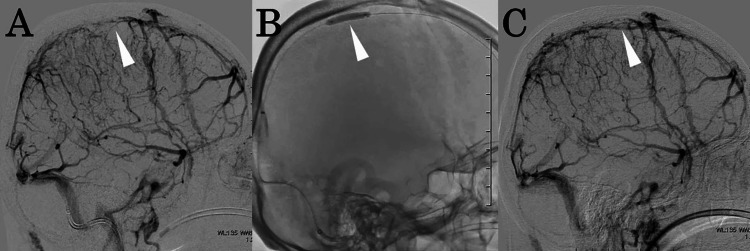
Cerebral angiography findings before and during balloon angioplasty (A) Pre-treatment angiographic findings showing a segment of poor contrast enhancement in the superior sagittal sinus (white arrowhead). (B) The balloon (white arrowhead) was inflated and deflated at eight locations along the superior sagittal sinus while being gradually withdrawn. (C) Cerebral angiography showing recanalization of the superior sagittal sinus (white arrowhead).

**Figure 5:**
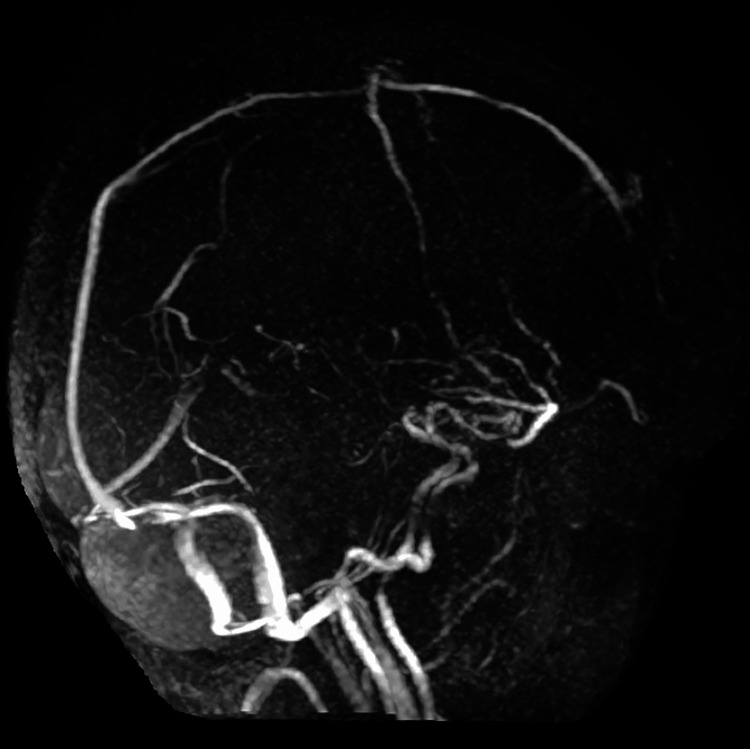
Follow-up magnetic resonance venography at 30 days No recurrence of CVST is observed. CVST, cerebral venous sinus thrombosis

We reached a diagnosis of acute CVST, and intravenous heparin was initiated at 500 units/h. On hospital day 3, the patient developed seizures, and a head CT revealed an intracerebral hemorrhage in the left frontal lobe (Figure 2). Heparin was discontinued, and levetiracetam was initiated. On hospital day 7, the patient experienced another seizure, and a new intracerebral hemorrhage was observed in the right parietal lobe on CT (Figure 3). Worsening of VST was suspected, and heparin therapy was resumed. However, the condition deteriorated progressively. Therefore, balloon angioplasty was performed on hospital day 10 to treat the VST. A 4F sheath was inserted into the left femoral artery, and a catheter was placed in the right internal carotid artery for angiography (Figure 4A). A 6F guiding sheath was inserted into the left femoral vein and advanced to the right sigmoid sinus. A Navien intermediate catheter (Medtronic, MN, USA) was used to navigate up to the posterior portion of the superior sagittal sinus. A percutaneous transluminal angioplasty balloon (SHIDEN 4×20 mm; KANEKA, Tokyo, Japan) was carefully advanced beyond the level of the coronal suture and gradually pulled back, with inflation and deflation performed at eight points along the superior sagittal sinus (Figure 4B). The inflation pressure was 0.8 MPa; each inflation was maintained for 30 s. This procedure was repeated twice, resulting in successful recanalization of the superior sagittal sinus (Figure 4C). Postoperatively, no new intracerebral hemorrhage was observed. Intravenous heparin infusion (500 units/h) was continued, combined with strict blood pressure control. The activated partial thromboplastin time was maintained at approximately 1.5-2 times the normal range, after which anticoagulation was transitioned to oral warfarin. Follow-up magnetic resonance venography 30 days later confirmed no recurrence of CVST (Figure 5). After the patient’s condition stabilized, he was transferred to a rehabilitation hospital on hospital day 38 with residual right hemiparesis and a modified Rankin Scale (mRS) score of three. Informed consent was obtained from the patient for the publication of this case report.

## Discussion

Known risk factors for CVST include oral contraceptive use, hormone therapy, antiphospholipid syndrome and other acquired thrombophilias, malignancies, autoimmune diseases, hereditary thrombophilias, head trauma, and dehydration [2]. In addition, an increased incidence of CVST has been reported after COVID-19 infection and vaccination [6]. We report a rare case of progressive superior sagittal sinus thrombosis in a patient with no identifiable risk factors, successfully treated with endovascular balloon angioplasty alone. The patient, a 68-year-old man, presented with right upper limb weakness and motor aphasia. Despite initial anticoagulation therapy, his condition progressively worsened, with the development of bilateral intracerebral hemorrhages. Given the deterioration and imaging findings of worsening thrombosis, balloon angioplasty was performed as a rescue endovascular intervention on hospital day 10, resulting in successful recanalization and stabilization. This case is notable for the following reasons: (1) CVST presented with progressive neurological deterioration and intracerebral hemorrhages, (2) there were no identifiable underlying risk factors or comorbidities, and (3) balloon angioplasty alone, without thrombectomy or thrombolysis, led to a favorable outcome. To our knowledge, this is an exceptionally rare report of CVST with such a clinical background being successfully treated using balloon angioplasty alone.

Several case reports have described successful endovascular interventions, including mechanical thrombectomy and balloon angioplasty, in patients with severe or refractory CVST [4,5]. These reports suggest that endovascular management may be a viable option in select cases. In line with these findings, our case adds further support to the notion that endovascular treatment can be a practical and effective alternative, especially when medical therapy fails or the patient exhibits poor prognostic features.

The prognosis of CVST is generally favorable if treatment is promptly initiated after early diagnosis [7]. However, approximately 10-15% of patients experience death or dependence, indicating that some cases follow a severe clinical course [2]. Known poor prognostic factors include advanced age, coma (Glasgow Coma Scale <9), male sex, intracerebral hemorrhage, and malignancy, all associated with a two- to three-fold increased mortality risk [8]. Despite the importance of timely management during the acute phase, there is a lack of solid evidence supporting the efficacy of endovascular treatment for CVST, and current knowledge is primarily based on case reports and limited clinical experience [2,4,5]. Despite reports of >90% recanalization rates with endovascular intervention, the associated complication rate remains relatively high (10.3%) [9]. Among these complications, intracranial hemorrhage is the most frequently observed, occurring in approximately 2.9% of cases [9]. One of the reasons for the relatively high complication rate is the risk of penetrating fragile cortical veins during guidewire manipulation in endovascular procedures. In addition, the large thrombus burden within the venous sinus may necessitate multiple retrieval attempts during thrombectomy, potentially increasing the risk of venous dissection and endothelial injury. Thus, the optimal timing for endovascular intervention in CVST remains unclear. Moreover, it remains unclear which endovascular approach, thrombolytic therapy, balloon angioplasty, mechanical thrombectomy, or a combination of these, is most effective [2]. There are also case reports suggesting that stent placement may be effective in selected cases of CVST [10].

In the present case, the patient exhibited several poor prognostic factors, including coma (GCS <9), male sex, and intracerebral hemorrhage. Despite medical management, the condition progressively deteriorated, necessitating endovascular intervention. Given the 10-day period since symptom onset and repeated hemorrhagic infarctions, mechanical thrombectomy posed a high risk of intracerebral hemorrhage [11]. Therefore, balloon angioplasty, an approach that imposes relatively low mechanical stress on the vessel walls, was selected. Balloon inflation flattened and disrupted the thrombus within the sinus, allowing restoration of the normal vessel diameter. In this case, a total of 16 inflation-deflation cycles were carefully performed at multiple sites along the stenotic segment of the superior sagittal sinus. This meticulous technique and prolonged procedure time may have contributed to successful recanalization. Moreover, the restoration of venous outflow combined with continued anticoagulation therapy likely contributed to the recovery of the bridging cortical veins. As a result, no further hemorrhagic infarction occurred, and the patient had a relatively favorable clinical course without complications. Accordingly, balloon angioplasty was considered a safe and effective therapeutic option for progressive CVST, even in cases with delayed presentation after symptom onset.

## Conclusions

We report a case of progressive CVST successfully treated with balloon angioplasty. This treatment may be a valuable option for cases where symptoms worsen and conventional treatment proves difficult. Balloon angioplasty imposes relatively low mechanical stress on the vessel walls, minimizing the risk of complications such as intracranial hemorrhage. In this case, the careful execution of multiple cycles of balloon inflation and deflation likely contributed to the successful recanalization.

While balloon angioplasty was a safe and effective treatment in this case, careful patient selection is crucial. Moving forward, further analysis of similar cases is needed to clarify the efficacy and safety of endovascular treatment for CVST.
